# Patient perspectives on stress after ICU and a short primary care based psychological intervention – results from a qualitative sub‑study of the PICTURE trial

**DOI:** 10.1186/s12875-024-02698-6

**Published:** 2025-01-15

**Authors:** Antina Beutel, Linda Sanftenberg, Chris M. Friemel, Robert Philipp Kosilek, Maggie Schauer, Thomas Elbert, Ulf-Dietrich Reips, Tomke Schubert, Sabine Gehrke-Beck, Konrad Schmidt, Jochen Gensichen, for the PICTURE study group, Antina Beutel, Linda Sanftenberg, Chris M. Friemel, Maggie Schauer, Thomas Elbert, Ulf-Dietrich Reips, Tomke Schubert, Sabine Gehrke-Beck, Konrad Schmidt, Jochen Gensichen, Christine Adrion, Matthias Angstwurm, Antje Bergmann, Gerhard Bielmeier, Andrea Bischhoff, Ralph Bogdanski, Franz Brettner, Christian Brettschneider, Josef Briegel, Martin Bürkle, Johanna Dohmann, Peter Falkai, Thomas Felbinger, Richard Fisch, Hans Förstl, Benjamin Fohr, Martin Franz, Patrick Friederich, Jürgen Gallinat, Herwig Gerlach, Andreas Güldner, Hanna Hardt, Christoph Heintze, Andreas Heinz, Axel Heller, Christian von Heymann, Petra Hoppmann, Volker Huge, Michael Irlbeck, Ulrich Jaschinski, Dominik Jarczak, Stefanie Joos, Elisabeth Kaiser, Melanie Kerinn, Frank-Rainer Klefisch, Stefan Kluge, Roland Koch, Thea Koch, Michelle Kowalski, Hans-Helmut König, Robert Philipp Kosilek, Peter Lackermeier, Karl-Ludwig Laugwitz, Tri Le, Yvonne Lemke, Achim Lies, Klaus Linde, Daniela Lindemann, Dagmar Lühmann, Stephanie May, Ludwig Ney, Jan Oltrogge, Wulf Pankow, Sergi Papiol, Maximilian Ragaller, Nikolaus Rank, Lorenz Reill, Hans-Peter Richter, Reimer Riessen, Grit Ringeis, Ann Rüchhardt, Gustav Schelling, Jörg Schelling, André Scherag, Martin Scherer, Antonius Schneider, Gerhard Schneider, Jürgen Schneider, Julia Schnurr, Susanne Schultz, Thomas G Schulze, Karin Schumacher, John Singhammer, Peter Spieth, Kerstin Theisen, Franka Thurm, Thomas Vogl, Karen Voigt, Andreas Walther, Dietmar Wassilowsky, Cornelia Wäscher, Regina Wehrstedt, Björn Weiss, Roland Weierstall-Pust, Marion Weis, Georg Weiss, Harald Well, Zöllner Christian, Bernhard Zwissler

**Affiliations:** 1 Institute of General Practice and Family Medicine, University Hospital, LMU Munich, Munich, Germany; 2 Institute of General Practice and Family Medicine, Charité – Universitätsmedizin Berlin, Berlin, Germany; 3 Department of Psychology, University of Konstanz, Konstanz, Germany

**Keywords:** Post-traumatic stress disorder, Post-intensive care syndrome, PICS, Narrative Exposure Therapy, Qualitative analysis, Mental health, Stressful memories

## Abstract

**Background:**

Approximately 20–25% of patients who survive medical treatment at an intensive care unit (ICU) develop post-traumatic stress symptoms. There is currently a gap in follow-up care for them. As part of the PICTURE study, general practitioners (GPs) carried out a brief interview-based intervention. The aim of this sub-study is to record the most distressing memories of ICU treatment from the patient’s perspective and their evaluation of a GP-based brief psychological intervention.

**Methods:**

Participants were recruited from the intervention group of the main PICTURE study using selective sampling. All of them had experienced an ICU stay with mechanical ventilation and severe organ failure in the previous two years. They were interviewed about their experience of psychological stress during their ICU stay and their retrospective evaluation of the intervention. Semi-structured, guideline-based telephone interviews were conducted for this purpose, processed, and analyzed using the structuring qualitative content analysis based on Mayring.

**Findings:**

When asked *N* = 8 patients about the most stressful memory of their stay at ICU, the main themes were helplessness, pain, fixation, inability to communicate and sleep disturbances. The question of amnesia regarding the stay in the ICU was answered affirmatively by half of the interviewees but was not experienced as stressful. The brief trauma-focused intervention carried out by their GPs was well received by all respondents.

**Conclusions:**

The interviewees confirm that aversive traumatizing experiences are often associated with intensive care treatment and reinforce each other. These are due to the treatment setting but should be reduced wherever possible. In view of chronification and the lack of specific follow-up treatment options for these patients and the long waiting times for psychotherapy, the implementation of low-threshold treatment options by GPs appears to be ideally suited to closing this gap in care, particularly for patients with mild to moderate symptoms of a post-traumatic stress disorder.

**Trial registration:**

The main trial was registered at ClinTrials gov (NCT03315390) and at the German Register of Clinical Trials (DRKS, DRKS00012589) on 17/10/2017.

**Supplementary Information:**

The online version contains supplementary material available at 10.1186/s12875-024-02698-6.

## Background

### Post-intensive care syndrome (PICS) and post-ICU PTSD

The symptom complex of post-intensive care syndrome (PICS) refers to long-term physical, psychological and cognitive impairments that are related to intensive care treatment over time [24]. Approximately 60% of survivors are affected [2, 35]. However, the difficult to determine due to heterogeneous study designs, and the International Statistical Classification of Diseases and Related Health Problems ICD 11 does not yet have a diagnosis code for PICS. One of the sequelae of PICS is post-traumatic stress disorder (post-ICU PTSD). It is assumed that 20 to 25% of survivors of intensive care treatment develop post-ICU PTSD [25]. The prevalence is also expected to increase as a result of the Covid-19 pandemic [19].

To date, it has not been sufficiently clarified to what extent the type of memories of an intensive care unit (ICU) stay influence the severity of post-ICU PTSD symptoms [11, 18, 22].

### Status of therapeutic resources, gaps in care

Although medical treatment at an ICU is often accompanied by phases of great helplessness and fear and thus fulfils the subjective criterion of potentially traumatizing life events, there are hardly any aftercare programs for PICS sufferers in Germany. However, functional limitations in everyday life and comorbidities already manifest themselves in subsyndromal PTSD [36]. Even once the diagnosis has been made and a need for treatment has been identified, there is usually a long wait of three to nine months for a psychotherapy place [3]. In addition to the often severely impaired quality of life of those affected, the loss of productivity due to absence from work and the increased use of the healthcare system are also of socio-economic interest [13].

## Narrative Exposure Therapy NET

The implementation of trauma-focused narrative exposure therapy (NET) in the GP context of primary care was first tested as a short version as part of the PICTURE study. Three essential therapy components of NET were condensed for the brief GP intervention (psychoeducation, lifeline, narration of the trauma) in order to enable a structured and detailed processing of stressful memories [8]. The subsequent localization of the traumatic event in the biographical context has been shown to reduce psychological stress [29, 30].

The main aim of this qualitative accompanying study is to record the psychological stress peaks of survivors of intensive care treatment. All interviewees had received a NET intervention from their GP as part of the PICTURE study and commented on this during the interview. Subjective assessments of the benefits of this intervention are also reported but are not the main focus of this study as these results are published elsewhere [28].

## Methods

### PICTURE main trial

The PICTURE study is a DFG-funded multicenter study with sites in Munich (Ludwig-Maximilians-Universität) and Berlin (Charité Universitätsmedizin) that was conducted in the period from 2017 to 2024 [9]. The aim of this randomized controlled trial is to improve the care situation of patients with post-ICU PTSD by involving general practitioners. For patients who show mild to moderate stress based on the inclusion criteria, GPs carry out a brief trauma-focused intervention based on Narrative Exposure Therapy (NET) in three 45-minute sessions. They are trained by psychologists from the PICTURE team and supervised during the course of the intervention [7]. The PICTURE study was approved by the ethics committee of LMU Munich, and all study participants gave written informed consent.

Patients were 18–85 years old and had experienced an intensive care stay with mechanical ventilation and severe organ failure in the previous two years (SOFA score/ Sequential Organ Failure Assessment score ≥ 3). Patients who suffered from severe depression, acute suicidal tendencies or addictions, who were receiving neuroleptic, antiepileptic or anticholinergic long-term medication as part of an underlying mental illness or who were already participating in psychological trauma therapy were excluded from participation in the study.

### Recruitment and data collection for the sub-study

To represent the widest possible range of the sample (maximum variance), *N* = 10 participants from the NET intervention group (*N* = 160) were selected from the database of the main PICTURE study using selective sampling. The selection was based on a balanced distribution of gender, age, educational background, size of place of residence and admission diagnosis (see Table 1).

All respondents had already received the scheduled three NET sessions.

Semi-structured guided telephone interviews were conducted and -digitally recorded. An interview protocol was created for each interview.

The interview guide was created deductively (Additional file 1). The theoretical framing was based on study results on the connection between the severity of memories of the intensive care unit stay and the course of mental health.

All interviews were conducted by AB at home by telephone. There were no other people in the room during the interviews. The interviewees knew at the time of the interview that the interviewer was a master’s student of psychology and was conducting the interview for a study accompanying the main PICTURE study. They had no knowledge of her previous professional experience as an intensive care nurse. Helfferich’s manual [16] was used to prepare for conducting the interviews and to reflect on the role of the interviewer. The resulting findings were implemented for conducting the interviews.

The data collection was closed after all participants in the sample had been interviewed.

The participants did not receive any feedback on the content analyses following the interview.

The survey was conducted as part of the preparation of a master’s thesis and was therefore very limited in terms of time. For this reason, it was not possible to carry out a pilot phase.

The reporting follows the reporting guidelines of consolidated criteria for reporting qualitative research COREQ [33] (Additional file 2).

### Data analysis

First, the audio-digitally recorded telephone interviews were transcribed verbatim. The subsequent initiating text work and all further steps to raise the level of abstraction as well as the analysis of the data were carried out by AB in the sense of a structuring qualitative content analysis based on Mayring [21] using MAXQDA software (VERBI Software). Deductive and inductive approaches were combined during category development.

For quality assurance, a constant collegial exchange took place with the PICTURE teams of both study centers and in qualitative research circles (Berlin Methods Meeting for Qualitative Research). In addition, two exemplary interviews were cross coded by TS. Those codes that showed insufficient agreement were discussed together and the coding tree was revised again. All suggestions for better intersubjective comprehensibility of the code definitions were incorporated.

## Findings

The interviews were conducted in the period from 20.04.2021 to 24.06.2021 with a duration of 37 to 70 min (M = 52 min.). Of the ten people invited to the interview, two had to be excluded (*n* = 1 due to lack of cooperation, *n* = 1 due to time constraints). Table 1 describes the demographic characteristics of the interviewees. There were four female and four male post-ICU patients with an average age of 60.9 years. The reasons for treatment in the ICU varied widely, with the most common admission diagnosis being a liver or lung transplant. More than half of the interviewees had a high level of education and most lived in small towns.

**Table 1:** Patient characteristics (*n* = 8)

Characteristic		Number [*n*]
**sex**	male female	4 4
**age**	40–50 years 51–60 years 61–70 years	2 3 3
**educational history**	secondary schools intermediate maturity high school diploma	2 1 5
**Place of residence**, number of inhabitants	≤ 10.000 10.001 to 25.000 25.001 to 1.5 Mio	3 4 1
**admission diagnosis**	cardiac surgery neurosurgical intervention polytrauma sepsis transplantation (liver or lung)	2 1 1 1 3

The answers to the main question about the most stressful memories of the intensive care unit could be allocated to three categories: The experiences of helplessness, pain and restraint, aspects of restricted and denied communication and sleep disturbances.

In addition, the question about amnesia regarding the intensive care stay was also answered and presented below. Respondents also commented on their experiences with the NET intervention. These are only reported briefly, as the results have been published in detail elsewhere [28].

*Note for the following presentation of the results: “(…)” means a pause in the narrative flow*,* “[…]” means shortening the quote.*

### Memories of the intensive care unit (ICU), stress peaks

1. Experiences of helplessness, pain and fixation.

The interviewees reported phases of great helplessness, fear and loss of control due to restraint measures. This makes it clear that subjective criteria of potentially traumatizing life events can go hand in hand with the intensive care unit setting.

*“Something like helplessness. (…) That was the first feeling when I was awake again*,* so I was totally*,* yes*,* practically like a baby. So*,* at first I couldn’t do anything at all (…)” (B8: 7).*

*“[…] for me*,* this hospital consisted only of pain. (…) I looked at a white ceiling and it was just pain.” (B6: 119)*.

Two patients reported the threat or use of restraint:

*“So*,* when I woke up there in the middle of the night for the first time*,* I just realized that (…) my hands were fixed and […] that was somehow (…) already […] stressful in the situation.” (B5: 18)*.

2. Aspects of restricted and denied communication.

Due to intubation, verbal communication is not possible. In addition, staff sometimes refuse the wish to communicate in writing. It is perceived as stressful to be ignored in terms of communication.

The stress potential of not being able to communicate verbally is exacerbated by the aspects of fixation mentioned above: communication is not possible verbally, through gestures or in writing and reinforces feelings of great helplessness and loss of control. This deprives people, as relational beings, of nothing less than the satisfaction of a basic need.

*“[…] I also had the feeling that the people who were in there somehow didn’t talk to me at all*,* but rather talked about me*,* and that was somehow (…) already […] stressful in the situation*,* because I wanted to make myself understood somehow*,* but of course had the intubation tube in*,* couldn’t speak*,* then I wanted to somehow signal that I wanted to write something down*,* and they then noticed that and then said*,* well*,* they can’t do that anyway and then they always talked about me like that. […] And (…) somehow I would have liked them to talk to me a bit differently.” (B5: 18)*.

3. Sleep disturbances.

When answering the questions about sleep quality, it becomes clear that two almost irreconcilable aspects come together in an intensive care unit: On the one hand, restful sleep is a basic need for every person. On the other hand, the intensive care unit setting is characterized by constant vigilance to be able to react immediately to save lives. The monitoring cables, the ventilation and infusion tubes and the constant hustle and bustle on the ICU prevent restful sleep. Post-operative exhaustion exacerbates this problem.

*“I [have] not been able to sleep for a few days because […] (…)*,* of course you’re connected to equipment*,* that’s on top of everything else.” (B3: 12)*.

*“So*,* (…) then the night came again*,* the next night*,* I didn’t sleep again*,* the other patient was very restless*,* the one with the stroke*,* and then they operated on another patient in the intensive care unit*,* he came back again*,* then there was actually a whole team of doctors standing there for a relatively long time and I couldn’t sleep again.” (B3: 7)*.

### Statements on amnesia

Due to sedation, survivors of ICU treatment often have no or distorted memories of their ICU stay. This can have an impact on the psychological processing of the experience. Four of the eight interviewees answered the question about amnesia regarding the ICU stay affirmatively.

*“So*,* the first intensive care stay*,* as I said*,* that is completely missing (…)*.

*I: Does it bother you that this is missing?*

*B4: It's not dramatic, no. I don't find that bad." (B4: 62–64)*

On the other hand, two of the interviewees reported that amnesia had a positive aspect for them because it spared them aversive memories:

*"[...] I'm glad I wasn't awake before because [...] I had a fixator in my hand because I broke my ulna and radius, and then after four days they took the fixator out and put plates in my hand, (...) but I didn't realize all that." (B6: 147)*

### Assessment of the GP intervention

All interviewees reported that they had subjectively benefited from the sessions. Psychoeducational elements and being allowed to talk in a professional setting appear to be powerful factors here. The validation of emotions has the effect of normalizing negative feelings, and the professional complicity of the GP has a relieving function:

*“But if you tell someone else*,* practically an outside person*,* it just does you good*,* you’ve let off a bit of baggage*,* and if you’re then not made out to be completely “gaga” by this person either*,* then that’s helpful.” (B8: 165)*.

In an anxiety-free space, the symptoms can be normalized:

*“And above all […]*,* if there is such a narrative therapy*,* then of course you also know: you’re not alone in having such problems*,* because otherwise there wouldn’t be such a study in the first place. And I think that helps tremendously.” (B8: 165)*.

Without exception, all interviewees would explicitly recommend the intervention, even if they had not noticed any subjective improvement (one interviewee) or - as in the case of another interviewee - the stress caused by nightmares had only eased for a short time:

*“[…] because overall it was already very valuable for me to revisit this and also to become aware of it*,* and with that I was also able to (…) yes*,* process the whole thing better.” (B5: 126)*.

## Discussion

This exploratory qualitative study investigated the most distressing memories of intensive care treatment. Regarding stress peaks, the findings can be allocated to three categories: experiences of helplessness, pain and restraint, aspects of restricted and denied communication and sleep disturbances.

The interviewees also commented on the stress potential of amnesia regarding the ICU stay. They received a conversation-based brief intervention from their general practitioner as part of the PICTURE study. The assessment of this intervention is reported as a secondary finding.

The high burden of pain that is not symptom-controlled can have an impact on later mental outcome [10]. Inadequately treated or uncontrollable pain can increase the traumatizing potential of great helplessness and fear of death. In addition, there is often the need of restraint in phases of light sedation or in the wake-up phase to prevent accidental extubation or the removal of vital catheters due to uncoordinated patient movements [34]. The high traumatizing potential of restraint measures is well documented by many studies [6, 18]. Even if fixation is often unavoidable, it should be noted that the negative effects of helplessness, pain and fixation can reinforce each other.

Similarly stressful and reinforcing the traumatizing potential of the ICU is the restricted or even denied communication. The inability to communicate needs is associated with helplessness. The perceived lack of information is associated with the feeling of having no control [12]. Communication difficulties are associated by critically ill patients with the feeling of being “locked in” [32]. These results clearly show the enormous importance of patient-centered communication to limit the potentially traumatizing treatment conditions of the ICU. This is a complex challenge that must consider a wide variety of aspects such as teaching, training, multidisciplinary teamwork, family involvement, cultural diversity, technical support, and communication tools. The needs-adapted treatment concepts called for in terms of patient orientation require the presence of and contact with specialist staff [17]. However, their implementation seems questionable in view of the scarce personnel resources in intensive care units, especially as the situation has worsened dramatically due to the stress of the pandemic [4]. However, the statements made by the interviewees clearly show the importance of the presence of specialist staff in close proximity to the patient. Communication that conveys reassurance, safety, appreciation and information to patients is therefore necessary [12]. It is not a “dispensable comfort”, but the basis for a significantly improved mental course, as it can mitigate traumatic stress experiences [27]. Phases that are associated with great helplessness and lack of control and thus fulfil the subjective criterion of potentially traumatizing life events could thus be better classified by patients. To cut a long story short: Intensive care units keep people alive, communication and care keeps them in touch with life.

In terms of preventing poor mental outcomes, staff training is a high priority. Last but not least, the involvement of psychologists in the treatment team should be seriously considered. It has been shown that this supports the processing of traumatic experiences [26].

Sleep disturbances in intensive care units are often unavoidable. The causes are multifactorial: pain, environmental stimuli, healthcare-related interruptions, psychological factors, respiratory factors, and medications all influence sleep quality in the ICU. Nevertheless, poor sleep is considered to be one of the most common stresses experienced by critically ill patients [5]. Studies have shown that sleep disturbances promote the development of delirium [14, 23], which in turn is a relevant risk factor for post-ICU PTSD.

Sleep is a basic human need, as is the ability to communicate, the need to maintain control over the situation and the need to be free of pain. Without these aspects, psycho-physical survival is impossible or only possible to a very limited extent. This also shows that the stress factors resulting from the intensive care unit setting can reinforce each other. It is undisputed that the main aim of intensive care treatment must be to save lives. However, this inevitably means that the environmental conditions bring with them high psycho-physical stress factors. This is a dilemma that can hardly be resolved. It is therefore all the more essential to reduce these stress factors wherever possible. The PADIS guideline recommends a multifactorial approach to facilitate restful sleep [5]. The necessity of training treatment teams should be emphasized at this point.

The question about amnesia regarding the intensive care stay was answered affirmatively by four of the eight respondents. Unexpectedly and in contrast to previous research findings [22], they reported that amnesia per se had no stress potential for them. According to the ‘early illness amnesia hypothesis’, amnesia in an early phase of illness has a positive correlation with the severity of traumatic stress after discharge [11]. In contrast, some of the interviewees even rated this as relieving, as it had spared them aversive experiences. It is possible that these statements should be seen against the background of a change in sedation management in recent years, which stipulates that benzodiazepines should only be used if strictly indicated, as they are considered to have a risk potential for poor mental outcome [1].

The NET brief intervention gave all respondents the opportunity to process the traumatic experiences of the ICU retrospectively with their GP. This enabled them to compensate for communicative deficits and injured feelings of safety. In this way, they were able to establish a new healing narrative for threatening experiences of the ICU. All of them rated this positively and the results of the PICTURE-main study show that this has a demonstrably relieving function [9]. The evaluation of barriers and opportunities for implementation of the intervention has already been published elsewhere [28].

Patients with post-traumatic symptoms often exhibit avoidance behavior despite limited everyday functionality and quality of life [31]. They therefore rarely seek support from psychotherapists. As the GP practice is usually the most important point of contact for these patients, the diagnosis of post-ICU PTSD and initial low-threshold treatment for mild to moderately stressed patients should take place in this setting. Psychological support in coming to terms with the experience has a positive impact on quality of life after a critical illness. Long waiting times for psychotherapy places are bridged and the experience of stress is quickly reduced. As a result, those affected regain their ability to cope with everyday life sooner. This needs-adapted approach has already proven successful in other areas of mental health care [15, 20].

### Strengths and weaknesses of our study

This exploratory study is an encouraging basis for further research in this field.

A selection bias can be assumed in the recruitment of interviewees, as presumably only those patients who are generally open to trauma therapy gave their consent to the PICTURE study. A social-accepting bias cannot be ruled out either. However, even if the participants have agreed to be interviewed, they are not obliged to disclose their relevance system. The interviewer’s implicit expectation of openness on the part of the participant can therefore be violated [16]. In addition, the context dependency of the narrative means that it can only ever be a snapshot (ibid.).

As the interview guide was carried out as part of the preparation of a master’s thesis, the available time frame was therefore very limited. For this reason, no pilot phase could be carried out and the criterion of saturation is limited with a sample size of *N* = 8.

Furthermore, the results can hardly be transferred to other vulnerable groups, such as patients with dementia or children and adolescents. Further studies are required here.

The NET brief intervention in GP practices is a promising approach that uses the existing healthcare system to help meet the need for low-threshold mental health support. If the NET short version could be integrated into standard care, practical exercises and supervision by NET specialists should be an integral part of GP practice.

This approach is probably not sufficient for highly traumatized patients, who must be given prompt access to specialized therapists.

## Conclusion

Even before the coronavirus pandemic, the incidence of post-ICU PTSD was conservatively estimated at around 25% of ICU survivors. The figures are expected to rise significantly in the future. The interviewees confirm that aversive traumatizing experiences are often associated with intensive care treatment. These are due to the treatment setting but should be reduced wherever possible. Regarding the limited everyday functionality of those affected and the avoidance of chronification of symptoms, concepts for reducing the long-term consequences of critical illness should be expanded. Primary care clinicians are faced with the major challenge of offering evidence-based therapy for the treatment of post-traumatic symptoms. As the therapy offered by the NET short version is low threshold for patients and practitioners alike, integration into standard care should be considered. This also has an economic urgency, not least in view of the social burden of trauma-related costs.

## Supplementary Information

Supplementary Material 1

Supplementary Material 2

## Data Availability

Data is provided within the manuscript or supplementary information files.

## Abbreviations

PICS: Post intensive care syndrome
PTSD: Post-traumatic stress disorder
NET: Narrative Exposure Therapy
GP: General practice/ general practitioner
ICU: Intensive care unit
