# Supplementary material for: Patient perspectives on stress after ICU and a short primary care based psychological intervention – results from a qualitative sub‑study of the PICTURE trial
Source: BMC Prim Care. 2025 Jan 15;26:12. doi: 10.1186/s12875-024-02698-6 (PMC11734548; doi:10.1186/s12875-024-02698-6)
Supplement: Supplementary file 1 — Supplementary Material 1 [file 12875_2024_2698_MOESM1_ESM.docx]

**Interview Guideline**

| **Main Questions** | **Storytelling prompt** | **Definition of the leading questions. Check: was this mentioned?** | **Concrete Inquiries** |
| --- | --- | --- | --- |
| **Memories of the ICU or experiences with the ICU stay.** | "What is the first memory that comes to mind when you think back to your stay at ICU?" | **Memories of the ICU:**  - Most stressful memory of the ICU stay ("stress peak")  - Is there amnesia regarding the intensive care stay?  - Are there delusional and distorted memories?  - Does the ICU memory trigger earlier traumatic experiences? | **Memories of ICU:**  - "What particularly bothers you about your memories of ICU?"  - "Do you miss the time you spent in ICU in your memory?"  - "Do you possibly only have incoherent or nightmarish memories of your time in the ICU?"  - "Do you have the feeling that since your stay in ICU, you have more stressful memories from earlier phases of your life?" |
| Statements on treatment success: What aspects are mentioned regarding **acceptance and effectiveness of NET?**  How do patients define success? | "What has changed for you since you completed talk therapy? How are you doing today?"  Finally, open-ended question: "What do you wish for the future in terms of your mental health"? | **Effectiveness of NET or changes since completion of therapy:**  **-** Has the overall QOL changed? How?  - What is the course of physical recovery? (control variable "somatic condition")  - What is the course of psychological recovery?  - Is the perception of suffering lower?  - Question about the social environment.  **Quality of memories:**  **-** What is the quality of memories of the intensive stay? Possibly fewer intrusions? Possibly fewer delusional and distorted memories? Possibly realistic classification?  - Dealing with amnesia during therapy: e.g. reconstruction through ICU diary, patient files or similar.  **Acceptance of the NET:**  **-** Is the therapy perceived as helpful overall? Subjective assessment of the long-term effectiveness of the therapy. | **Effectiveness of NET or changes since completion of therapy:**  - "How are you doing today in terms of your physical health?"  - "What in particular has contributed to your mental recovery?"  - "What stood in the way of your recovery?"  - "Has your daily life changed since therapy?"  - "Are you supported by those around you?"  **Quality of memories:**  - "After completing therapy, do you continue to be haunted by distressing memories of the ICU stay?"  - If yes: "How do you deal with this? What impact does it have on your daily life?"  - "Were you able to reconstruct the time of the missing memories through the therapy?"  **Acceptance of NET:**  - "Would you recommend the therapy to others?"  - "What do you wish for your mental health in the future?" |

*Note: In German language what-questions are close to how-questions.*
